# Cold physical plasma treatment optimization for improved bone allograft processing

**DOI:** 10.3389/fbioe.2023.1264409

**Published:** 2023-11-07

**Authors:** Maximilian Fischer, Emely Bortel, Janosch Schoon, Einar Behnke, Bernhard Hesse, Timm Weitkamp, Sander Bekeschus, Monika Pichler, Georgi I. Wassilew, Frank Schulze

**Affiliations:** ^1^ Center for Orthopaedics, Trauma Surgery and Rehabilitation Medicine, University Medicine Greifswald, Greifswald, Germany; ^2^ Xploraytion GmbH, Berlin, Germany; ^3^ ESRF: European Synchrotron Radiation Facility, Grenoble, France; ^4^ Synchrotron SOLEIL, Saint-Aubin, France; ^5^ ZIK Plasmatis, Leibniz Institute for Plasma Science and Technology (INP), Greifswald, Germany; ^6^ Cells + Tissuebank Austria Gemeinnützige GmbH, Krems an der Donau, Austria

**Keywords:** allografts, cancellous bone, cold atmospheric pressure plasma, plasma medicine, synchrotron radiation computed tomography, mesenchymal stromal cells

## Abstract

In musculoskeletal surgery, the treatment of large bone defects is challenging and can require the use of bone graft substitutes to restore mechanical stability and promote host-mediated regeneration. The use of bone allografts is well-established in many bone regenerative procedures, but is associated with low rates of ingrowth due to pre-therapeutic graft processing. Cold physical plasma (CPP), a partially ionized gas that simultaneously generates reactive oxygen (O_2_) and nitrogen (N_2_) species, is suggested to be advantageous in biomedical implant processing. CPP is a promising tool in allograft processing for improving surface characteristics of bone allografts towards enhanced cellularization and osteoconduction. However, a preclinical assessment regarding the feasibility of pre-therapeutic processing of allogeneic bone grafts with CPP has not yet been performed. Thus, this pilot study aimed to analyze the bone morphology of CPP processed allografts using synchrotron radiation-based microcomputed tomography (SR-µCT) and to analyze the effects of CPP processing on human bone cell viability and function. The analyzes, including co-registration of pre- and post-treatment SR-µCT scans, revealed that the main bone morphological properties (total volume, mineralized volume, surface area, and porosity) remained unaffected by CPP treatment if compared to allografts not treated with CPP. Varying effects on cellular metabolic activity and alkaline phosphatase activity were found in response to different gas mixtures and treatment durations employed for CPP application. It was found that 3 min CPP treatment using a He + 0.1% N_2_ gas mixture led to the most favourable outcome regarding a significant increase in bone cell viability and alkaline phosphatase activity. This study highlights the promising potential of pre-therapeuthic bone allograft processing by CPP prior to intraoperative application and emphasizes the need for gas source and treatment time optimization for specific applications.

## 1 Introduction

The treatment of large segmental bone defects is challenging and requires specialized surgical procedures and the employment of transplant materials. While many different natural and synthetic materials have been proposed for the treatment of large segmental defects, bone matrix itself still provides the most promising outcomes (Schulze et al., 2023). In this context, autologous bone grafts are still considered the gold standard (Sohn and Oh, 2019; Rothweiler et al., 2022). However, this procedure is limited in the quantity of graft material available and is accompanied by higher donor-site morbidity and infection due to the need for a second surgical site (Fillingham and Jacobs, 2016; O'Brien, 2011). Therefore, bone allografts have emerged as a favorable therapeutic option in bone regeneration (Campana et al., 2014; Baldwin et al., 2019). Allografts can be harvested from cadaveric sources or from material that is routinely discarded in orthopedic surgeries and thus available in higher quantities than autologous grafts. In orthopedic surgery, bone grafting is used in many indications such as septic and aseptic revision arthroplasty, spine surgery, bone plastics after tumor resections, or in case of osteotomies addressing skeletal deformities (Brydone et al., 2010; Gillman and Jayasuriya, 2021). The number of bone allografting procedures in Germany has increased by 74% over the last decade (Rupp et al., 2022). However, allogeneic bone grafts need to be deproteinized and sterilized to prevent immunogenic reactions and disease transmission (Fretwurst et al., 2014; Fillingham and Jacobs, 2016). This affects the ingrowth rates of allografts into the foreign bone, which are reported to range between 40% and 80%, depending on the exact type of pre-therapeutic graft processing such as decellularization by H_2_O_2_ or nuclear irradiation (Blokhuis and Lindner, 2008; Scheinpflug et al., 2018). The graft processing is necessary to reduce the bone grafts’ antigenicity and infection risks by, e.g., HIV, HBV, or HCV (Boyce et al., 1999; Ahmed et al., 2023). Depending on the type of these processing procedures, the graft materials’ cellularization potential, osteoconductivity, osteoinductivity, and mechanical properties can be affected negatively (DePaula et al., 2005; Lei et al., 2015; Mansor et al., 2023). Thus, innovative allograft processing strategies are needed to restore bone allograft characteristics and improve patient care. Cold physical plasma (CPP), characterized by the generation of a plethora of short-lived oxygen (O_2_) and nitrogen (N_2_) species around body temperature, could be a promising tool for innovation in manufacturing process of bone allografts (von Woedtke et al., 2020; Nonnenmacher et al., 2023). Thermal plasma spraying (>1,000°C) is already employed in the processing of the orthopedic metal implants to enhance osteoconductivity and for minimizing the risk of implant loosening (Killinger et al., 2021; Ratha et al., 2021). In contrast to thermal plasma spraying, CPP enables material processing at physiological biocompatible temperatures, which is important to maintain or improve osteoconductive properties of bone allografts (Weltmann et al., 2010; Nonnenmacher et al., 2023). Preclinical studies on CPP-processed titanium surfaces revealed that this surface processing improves biocompatibility by enhancing cell spreading and adhesion of musculoskeletal cells cultured on the respective material (Duske et al., 2012a; Canullo et al., 2013). Albeit plasma treatment is usually performed on surfaces, various examples from literature have demonstrated the feasibility of using CPP on 3D scaffolds with porous structures (Hadjizadeh and Doillon, 2010; Canal et al., 2016; Sardella et al., 2017). Furthermore, it was demonstrated that CPP processing of synthetic and natural porcine cancellous and cortical bone grafts resulted in increased protein absorption and cell adhesion of murine osteoblasts (Canullo et al., 2018). In this context, the gas source employed for CPP generation is critical for the biological effects observed and thus needs careful optimisation for each application scenario (Fischer et al., 2022; Nonnenmacher et al., 2023).

However, studies that focus on analyzing morphological parameters of bone are needed in the preclinical evaluation of pre-therapeutic bone allograft processing by CPP. It is important that the postprocessing of allogeneic bone grafts by CPP would not lead to changes in the biomaterial morphology that might subsequently alter its performance after implantation (Brydone et al., 2010). Therefore, this pilot study aimed to analyze 3D porous bone allografts after CPP processing by synchrotron radiation-based microcomputed tomography (SR-µCT). The parameters analyzed in terms of morphology were total tissue volume (TV), mineral volume (BV), bone surface (BS), bone porosity (Po), trabecular thickness (Tb.Th), and trabecular separation (Tb.Sp). In order to address clinical practicability, CPP processing was performed using the certified medical plasma jet kINPen and the clinically approved gas Argon (Ar). The effect of CPP application was investigated regarding bone cell viability and function by seeding human mesenchymal stromal cells (hMSCs) on CPP treated allogenic bone and investigated for their metabolic and alkaline phosphatase (ALP) activity. hMSCs are key players in bone regeneration and homeostasis (Scheinpflug et al., 2018).

In addition, different gas mixtures and treatment times were employed to optimise CPP application. We hypothesized that important morphological parameters are not adversely affected by CPP treatment of autologous bone grafts and that optimization of the plasma feed gas mixture can help increase bone cell viability and osteogenic function.

## 2 Materials and methods

### 2.1 Allogeneic bone preparation

The C + TBA provided human spongiosa plates for this research project treated with the proprietary Allotec process. Cylinders (6 mm height, 5 mm diameter) were punched out from these plates in the laboratories of the University Medicine Greifswald (Clinic for Orthopedics) under sterile conditions after overnight rehydration in phosphate-buffered saline (PBS). The cancellous grafts were collected from living donors that underwent hip replacement surgery. The tissue was cut into plates and treated in an ultrasonic bath with WFI to remove remaining fat, blood, donor cells, and pathogens. After sonication, the cancellous bone was washed with diethyl ether, ethanol in different concentrations, and 3% hydrogen peroxide to denature soluble proteins and remove any remaining pathogens. Lyophilization was performed to dry the tissue while retaining the morphological characteristics of the tissue, resulting in a water content of ≤10%. Gamma irradiation of the tissue was performed for final sterilization, and allogeneic bone samples were stored at room temperature until they were used in the experiment.

### 2.2 Exposure to cold physical plasma

The atmospheric pressure argon plasma jet kINPen (neoplas MED, Greifswald, Germany) was utilized. Details on its applications and safety profiles as well as construction and technical design were described previously (Bekeschus et al., 2016; Reuter et al., 2018). Argon gas (Air Liquide, Bremen, Germany; two standard liters per minute) was excited by a high-frequency electrode inside the jet to generate reactive O_2_ and N_2_ species (ROS and RNS) after expulsion to ambient air. The jet was attached to a *xyz*-motorized precision controller stage (CNC, Bremen, Germany), hoovering the gas plasma above the center of each well for the set time. Each scaffold was treated for 5 min with argon-derived CPP for SR-µCT analyzes. For cell culture experiments each scaffold was treated for one, three or 5 min with CPP derived from different gas mixtures. Ar and Helium (He) gas (Air Liquide, Bremen, Germany; two standard liters per minute) were used as carrier gases and mixed with up to 2% of either O_2_ or N_2_ gas (Air Liquide, Bremen, Germany; two standard liters per minute) to generate CPP.

### 2.3 Testing for sterility after exposure to CPP

To ensure the sterility of scaffolds after punching and CPP treatment, a test for bacterial growth was perfomed. Scaffolds were incubated in 5 mL LB medium for 24 h at 37°C on an orbital shaker. Tubes containing only LB medium served as negative controls while, CPP-treated scaffolds that were deliberately contaminated served as positive controls. After incubation time, 3 × 100 µL of LB media were transferred into a 96 well multititer plate for subsequent absorbance measurements at 600 nm using a multiplate reader (TECAN M200, Tecan, Switzerland).

### 2.4 Synchrotron radiation-based microcomputed tomography

Samples were scanned using SR-µCT at the Anatomix beamline at the Synchrotron Soleil in Paris at a naïve state (t1) and after surface CPP treatment (t2) (Weitkamp et al., 2017; Weitkamp et al., 2022). The central energy of the polychromatic (“pink”) X-ray beam was set to 40 keV and the voxel size was 3.07 µm. Each scan was done with a slight offset of the rotation axis with respect to the detector field of view, to increase the diameter of the reconstructed volume. For each scan, between 3,000 and 4,000 projections, depending on the rotation axis offset, were acquired with 50 ms exposure per projection. Tomographic reconstruction was performed using a Python-based configuration tool written at SOLEIL and calling the reconstruction program PyHST2 developed at the European Synchrotron Radiation Facility (ESRF) (Mirone et al., 2014). A phase-retrieval filter of Paganin type was used, with the parameter “Paganin length” in PyHST2 set to 138 µm (Paganin et al., 2002).

### 2.5 Data processing and analyzes

A registration of regions of interest of the t1 and t2 volumes was done using the affine registration plug-in installed in Avizo 2019.3 (Thermo Fisher Scientific, Waltham MA, United States). The mineralized bone was segmented using a workflow implemented in Matlab 2022a (The MathWorks, Natick MA, United States). In a first step, the gray value datasets were binarized using a simple Otsu threshold. The obtained bone mask was dilated with a spherical structuring element with a radius of 3 voxel and applied to the gray value data. This masked dataset was thresholded using a 1.5-fold Otsu-value. To omit small broken bone fragments, the bone mask was eroded with a spherical structuring element with a radius of 3 voxel and each component was labeled. Only the biggest component was extracted and dilated with a spherical structuring element with a radius of 3 voxel to retain the original morphology. For each dataset TV (all voxels within the volume of interest), BV (voxels assigned as mineralized bone), Po (100×MV/TV), BS, Tb.Th (number of spheres within BV) and Tb.Sp (Number of spheres outside BV) were extracted. The BS was calculated by dividing the difference between the dilated and the eroded bone mask by 2 (Otto et al., 2022). The trabeculae were measured by a sphere fitting algorithm. The weighted means and corresponding standard deviations were calculated for Tb.Th and Tb.Sp. To calculate the bone turnover, the t1 and t2 binarized datasets were coregistered and analysed such that theresorbed bone was assigned to a pixel value of 1, formed bone to a pixel value of 2, and constant bone had a pixel value of 3.

### 2.6 Cell seeding on allogenic bone scaffolds and investigation of viability and osteogenic function

Human bone-marrow derived mesenchymal stromal cells were isolated from metaphyseal bone marrow of one patient undergoing primary hip arthroplasty due to osteoarthritis as described previously (Fischer et al., 2022). Ethics approval (BB 160/20) was obtained from the local independent ethics committee (IEC) of the University Medicine Greifswald according to the World Medical Association Declaration of Helsinki. The donor (female, 49 years old) gave written informed consent.

The bone model used in this work is based on a previously published study (Schoon et al., 2020). In brief, hMSCs were cultured to passage two, trypsinated and seeded on CPP-treated or control bone scaffolds (three scaffolds in each group) with 6 mm height, 5 mm diameter. The scaffolds were placed in a 96-well ultra low attachement plate (Sigma Aldrich, St. Louis, MO, United States) for cell seeding. 3 × 10^5^ hMSCs suspended in 200 μL of cell culture media—DMEM low glucose (PAN Biotech, Aidenbach, Germany), 10% fetal bovine serum (FBS, Sigma Aldrich, St. Louis, MO, United States), 100 U/mL penicillin (Gibco, Waltham, Massachusetts, United States), 1% 100 μg/mL streptomycin (Gibco), 1% 2 mM L-alanyl-L-glutamine (GlutaMAX, Gibco, Waltham, Massachusetts, United States)—were pipetted onto the bone scaffolds followed by incubation for 2.5 h under standard cell culture conditions (37°C, 5% CO_2_). Next, the bone scaffolds were turned around by 180° to ensure uniform cell seeding. After another 2.5 h of incubation, the bone scaffolds were transferred to a 24-well flat bottom plate (Sigma Aldrich, St. Louis, MO, United States) and cultured under standard cell culture conditions in 1,000 μL of cell culture media for 7 days in total.

The cellular metabolic activity was measured on day four and day seven using a resazurin-based assay (PrestoBlue, Invitrogen, Waltham, Massachusetts, United States) according to the assay manual with slight modifications for 3D porous bone scaffols. In brief, a 1:10 mixture of assay reagent and cell culture media was prepared and 400 µL of that mixture were transferred into 2 mL Eppendorf tubes. The cell-laden scaffolds were introduced into the Eppendorf tubes followed by 1 h incubation at 37°C and 800 rpm rotation speed on a thermoblock. After incubation time, the scaffolds were removed from the tubes and 3 × 100 µL of the reagent mixture were transfereed into the wells of a 96-well multititer plate for subsequent fluorescent measurements at 560/590 nm (Ex/Em) on a multiplate reader (TECAN M200, Tecan, Switzerland) according to the manufacturers’ instructions. After quantifying the metabolic activity at day 4 the expansion media was exchanged.

Following this, the cellular ALP activity was analyzed by colorimetric quantification as described earlier with modifications for 3D porous cell-laden scaffolds (Rakow et al., 2016). In detail, scaffolds were washed with 500 µL alkaline phosphatase (AP)- buffer in a 2 mL eppendorf tube. After AP-buffer was removed, a mixture of 250 µL AP- buffer with 250 µL p-nitrophenyl phosphate (pNPP) was added into the Eppendorf tube containing the scaffold, followed by 10 min. Incubation at 37°C and 500 rpm in a thermoblock. After incubation time passed, the reactions was stopped by adding 500 µL 1 M NaOH. For determination of ALP activity, 3 × 100 µL of the reagent mixture were transfereed into the wells of a 96-well multititer plate for subsequent absorbance measurements at 405 nm on a multiplate reader (TECAN M200, Tecan, Switzerland).

### 2.7 Statistical analysis

No samples or technical replicates were excluded from the statistical analyses. Sample size was not predetermined by statistical methods. Randomization was not applied, and the investigators were not blinded to group allocation during the experiment. GraphPad PRISM 9.0 (GraphPad Software inc. La Jolla, CA, United States) was used for exploratory statistical analysis and descriptive data plotting. Trabecular morphological characteristics were investigated by analyzing Tb.Th and Tb.Sp values of control and CPP treatment group at t1 using an unpaired Student’s *t*-test (*n* = 3). The morphological parameters, TV, BV, BS, and Po were determined at t1 and t2 for both the control and the CPP treated group and data from the two time points was compared and analyzed using a paired Student’s *t*-test (*n* = 3). A normal distribution of data regarding metabolic and ALP activity was assumed and a one-way ANOVA with Dunn’s *post hoc* test was performed (*n* = 6).

## 3 Results

### 3.1 Synchrotron radiation-based microcomputed tomography allowed for evaluation of trabecular morphology prior to CPP-treatment

**FIGURE 1:**
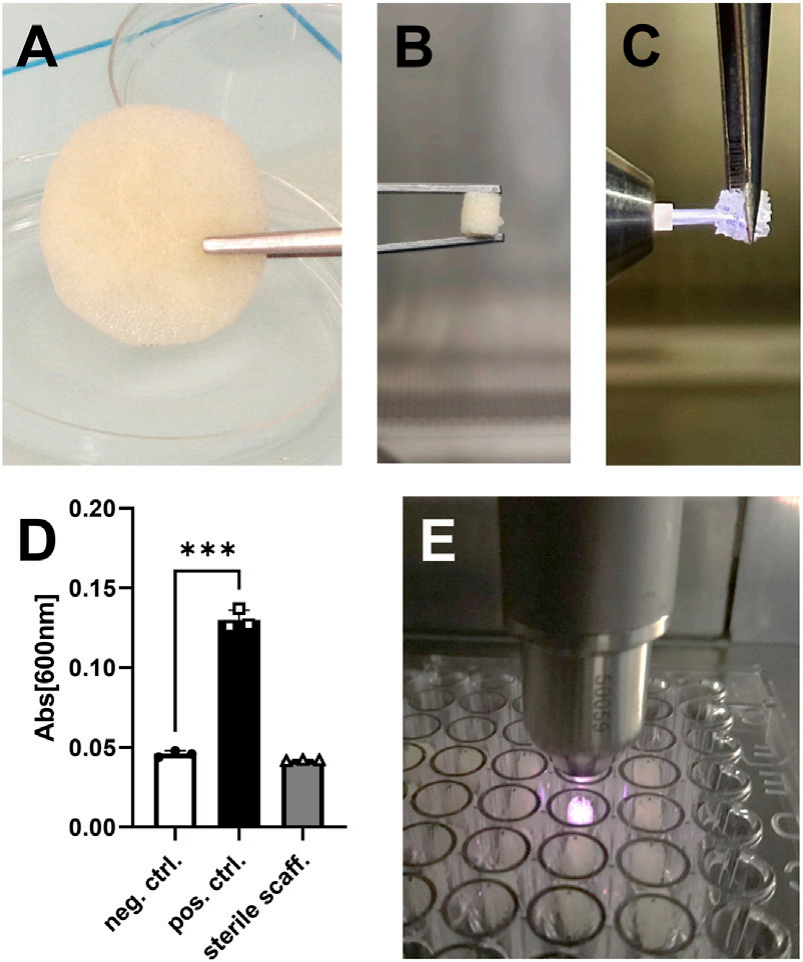
Preparation of allogeneic bone scaffolds. **(A)** Shown is a plate of trabecular allogeneic bone with a height of 5 mm after rehydration. **(B)** Cylindric pieces with a diameter of 6 mm were punched out from the plate. **(C)** The CPP flame is able to penetrate porous allogenic bone scaffolds. **(D)** No bacterial growth was found after 24 h, thus confirming sterility of the CPP treated scaffolds. **(E)** CPP treatment (t2) was performed in a multi-well plate using a clinical-grade device (kINPen). (D: Student’s *t*-test, paired, *n* = 3).

The porous 3D allogenic bone scaffolds for subsequent CPP treatment were prepared by punching 3D cylinders out of human spongiosa plates (Figures 1A, B). CPP treatment was found to be feasible by using a clinical argon plasma jet, as demonstrated by visible penetration of these porous scaffolds by the plasma flame (Figure 1C; Supplementary Video S1). To ensure the sterility of CPP treated allogenic bone, the absence of microbial contamination after incubation in bacterial growth medium for 24 h was confirmed (Figure 1D). For further experiments, the treatment with CPP was conducted using a automated XYZ-stage that facilitated reproducible and non-distructive treatment of multiple scaffolds under sterile conditions (Figure 1E).

**FIGURE 2:**
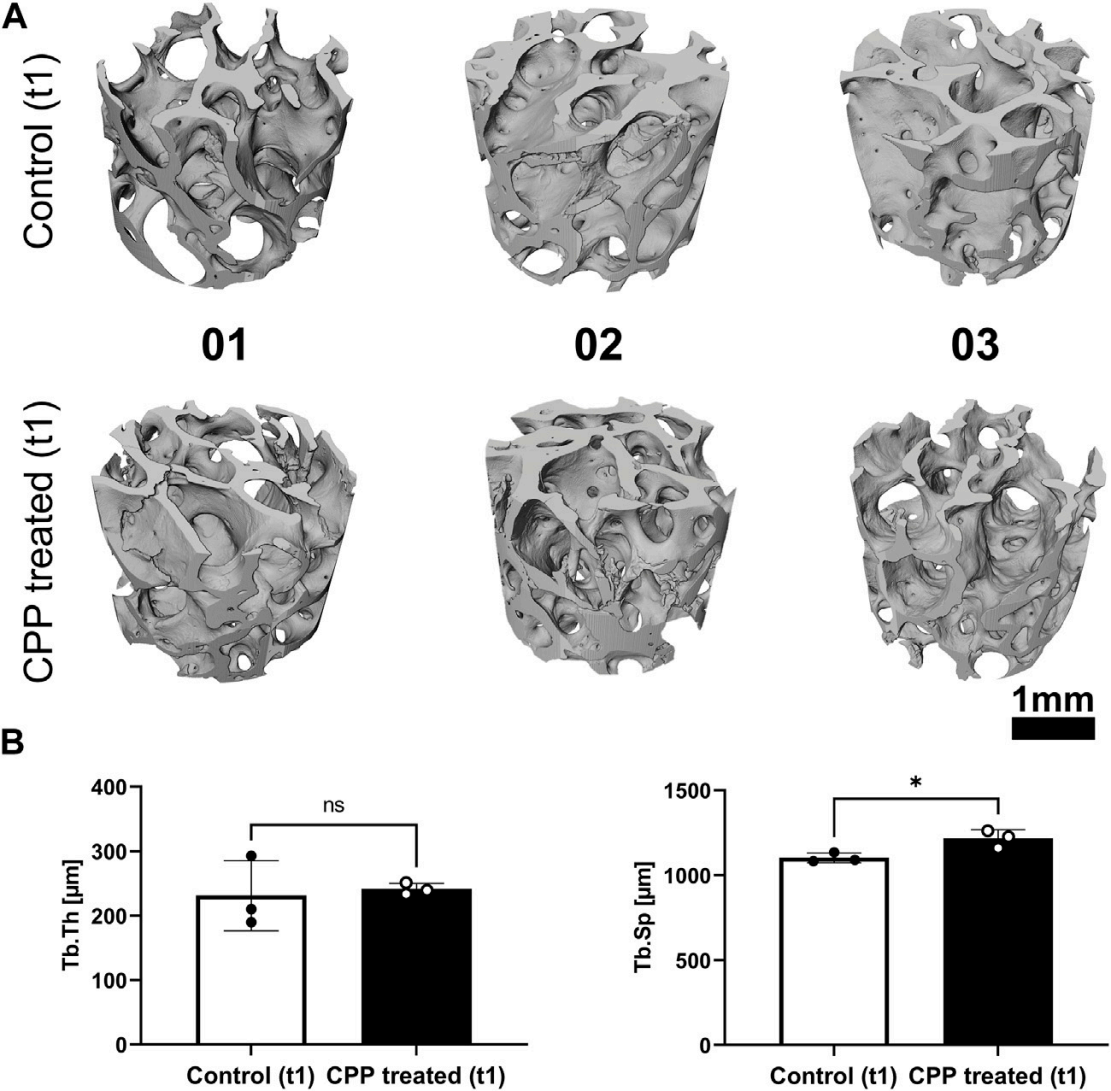
SR-µCT prior to CPP treatment for basic scaffold characterization. **(A)** Shown are the cropped regions of the reconstructed volumetric µCT data prior to CPP treatment (t1). **(B)** Tb.Th and Tb.Sp were determined prior to CPP treatment (t1) for all scaffolds. (Student’s *t*-test, unpaired, *n* = 3, *p < 0.05).

To evaluate the effect of CPP treatment on the morphology of allogenic bone grafts, three scaffolds were treated with carrier gas only to serve as controls and three scaffolds were CPP-treated (Figure 1). The carrier gas employed was Ar since it is approved for clinical use of CPP and thus represents relevance in the context of allogenic bone processing. 5 min plasma treatment was chosen as the maximum duration to investigate whether CPP application can influence the morphology of allogeneic bone grafts. To evaluate morphological parameters, the scaffolds were analyzed by SR-µCT before (t1) and after (t2) CPP treatment. A 3D reconstruction was done for all analyzed bone scaffolds at t1 (Figure 2A). In addition, Tb.Th and Tb.Sp were calculated for these scaffolds at t1. No significant differences between Tb.Th. Mean values were found between the CPP-treated group and their respective controls, while Tb.sp mean values were found to be significantly higher for the scaffolds that are meant to be treated CPP (Figure 2B). Albeit this parameter varies between the two groups, the actual difference of 113,9 ± 34,08 µm is rather small and still allows for comparison of these two groups. The scanned scaffolds were then used for control and CPP treatment and subsequent evaluation of additional morphologic parameters at t1 and t2.

### 3.2 Bone morphological parameters were not altered by cold physical plasma treatment

**FIGURE 3:**
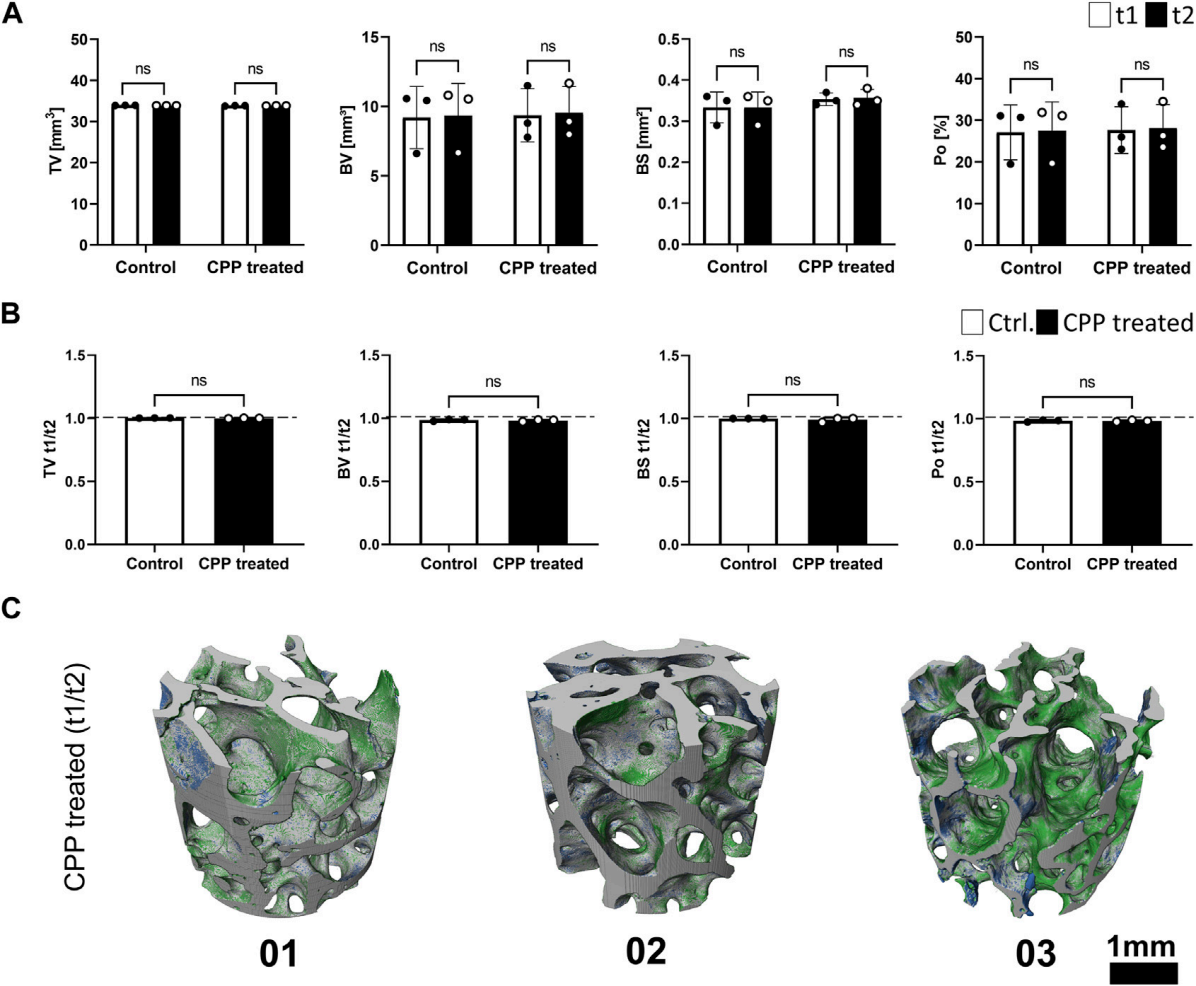
The influence of CPP treatment on morphological parameters of allogeneic bone. **(A)** Shown are morphological bone parameters for control and CPP treatment group. SR-µCT scans were performed prior to (t1, white bars) and after (t2, black bars) CPP treatment. **(B)** Shown are the values of the respective morphological parameters after normalization of t1 to t2. **(C)** Registration of volumetric reconstructions at t1 and t2 allows for the detection of TV loss (blue) and gain (green). The differences between t1 and t2 did not exceed the expected registration error. [**(A,B)** Student’s *t*-test, paired, *n* = 3].

After CPP treatment (t2), the TV was not altered in comparison to the initial bone volume, while comparison of the BV and BS between t1 and t2 indicated no changes for this parameter in response to CPP treatment (Figures 3A, B; Table 1). Comparing the BS between the two different time points for each group indicated no changes in the control group nor the CPP treated group (Figure 3B). The analysis showed small interindividual differences in Po in both study groups (Figure 3A; Table 1). There were no alterations in the Po comparing t1 and t2, and no CPP treatment induced changes (Figure 3B). Taken together, the quantification and comparison of main morphological bone parameters revealed no alterations between the two time points, indicating that CPP treatment did not induce alterations of these morphological bone parameters, within the limits of resolution of the imaging setup with a pixel size of 3 µm that was used for the study. Co-registration of t1 and t2 volumes of CPP treated scaffolds was conducted to quantify and visualise TV increase or decrease (Figure 3C). In summary, a 5 min long Ar-based CPP treatment did not induce measurable alterations in the investigated morphological parameters.

**TABLE 1:** Summary of morphological bone parameters.

Parameter	unit	Timepoint	Control					CPP-treated				
			01	02	03	Mean	SD	01	02	03	Mean	SD
Tb.Th	weighted mean ± stdev [µm]	t1	233.7 ± 73.4	215.1 ± 58.4	239.3 ± 57.1	229.4	12.6	190.0 ± 49.8	292.7 ± 71.8	210.3 ± 63.1	231.0	54.4
Tb.Sp		t1	1,134.9 ± 200.8	1,082.2 ± 183.0	1,090.4 ± 218.3	1,102.5	28.3	1,160.1 ± 201.0	1,262.0 ± 254.3	1,226.9 ± 218.8	1,216.3	51.8
TV	[mm³]	t1	33.9	34.0	34.0	33.9	0.0	33.8	33.9	33.9	33.8	0.1
		t2	33.9	33.9	33.9	33.9	0.0	33.9	33.8	33.9	33.9	0.0
BV	[mm³]	t1	6.6	10.6	10.4	9.2	2.2	7.8	11.5	8.8	9.4	1.9
		t2	6.7	10.8	10.5	9.3	2.3	8.0	11.7	8.9	9.5	1.9
BS	[mm²]	t1	0.3	0.4	0.4	0.3	0.0	0.4	0.4	0.3	0.4	0.0
		t2	0.3	0.4	0.4	0.3	0.0	0.4	0.4	0.3	0.4	0.0
Po	[%]	t1	19.5	31.1	30.7	27.1	6.6	23.0	33.9	26.0	27.7	5.6
		t2	19.7	31.9	31.1	27.6	6.8	23.6	34.5	26.3	28.1	5.7

### 3.3 Optimisation of CPP gas source to ensure survival and osteogenic function of bone forming cells

**FIGURE 4:**
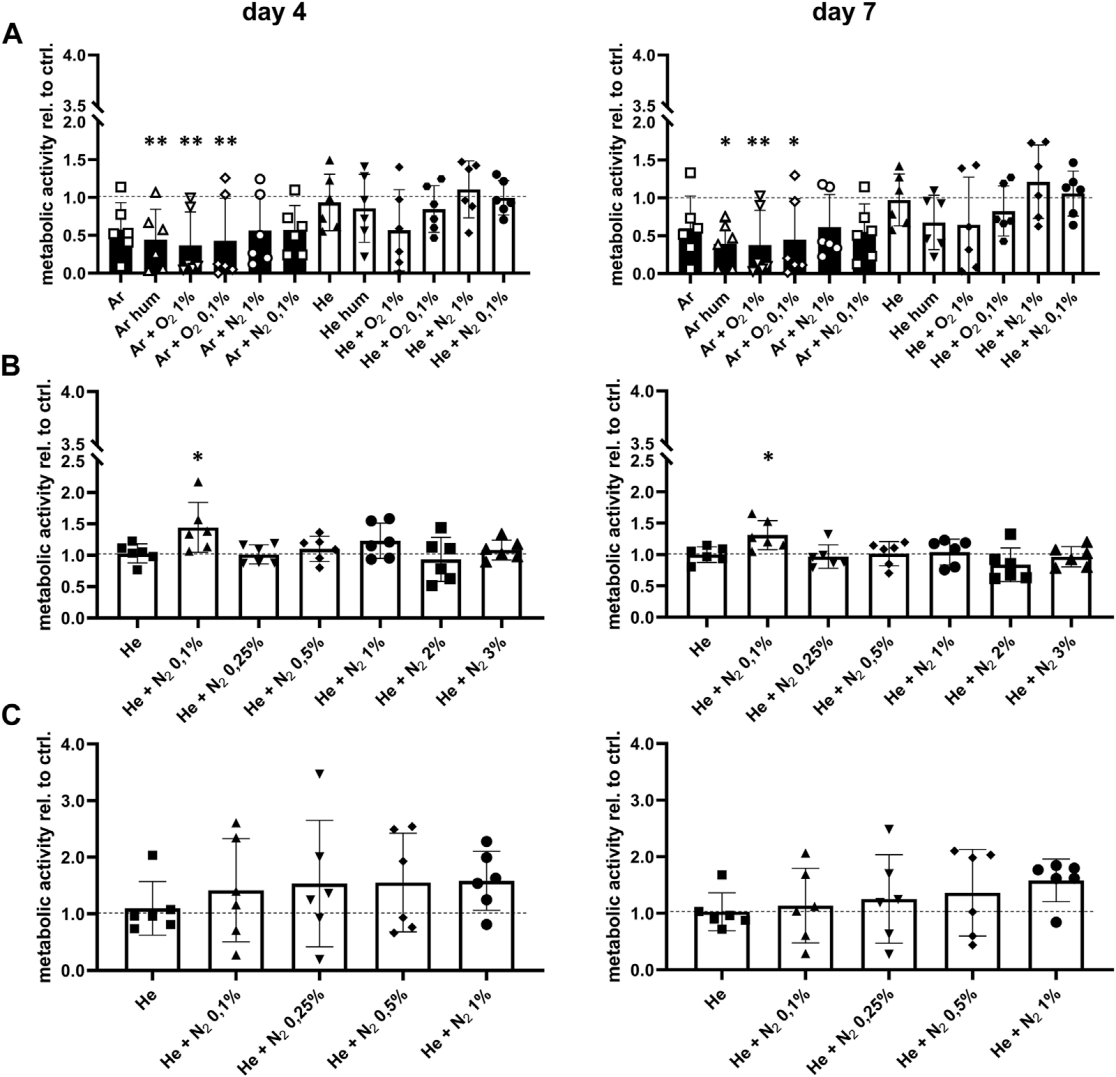
Optimisation of CPP gas source and treatment time prior to seeding with human mesenchymal stromal cells. **(A)** Scaffolds were treated by CPP using different carrier gases. The CPP treated scaffolds where then seeded with mesenchymal stromal cells, followed by investigation of metabolic activity after 4 and 7 days **(B)** Carrier gas sources were refined and CPP treatment time was prolonged to 3 min. After subsequent cell seeding, metabolic activity was determined after 4 and 7 days. **(C)** Based on previous results, CPP treatment time was prolonged to 5 min while only 5 carrier gases were tested. Metabolic activity of mesenchymal stromal cells seeded onto the CPP-treated scaffolds was determined after 4 and 7 days. [**(A–C)** one-way ANOVA, multiple comparisons, *n* = 6, *p < 0.05, **p < 0.005].

**FIGURE 5:**
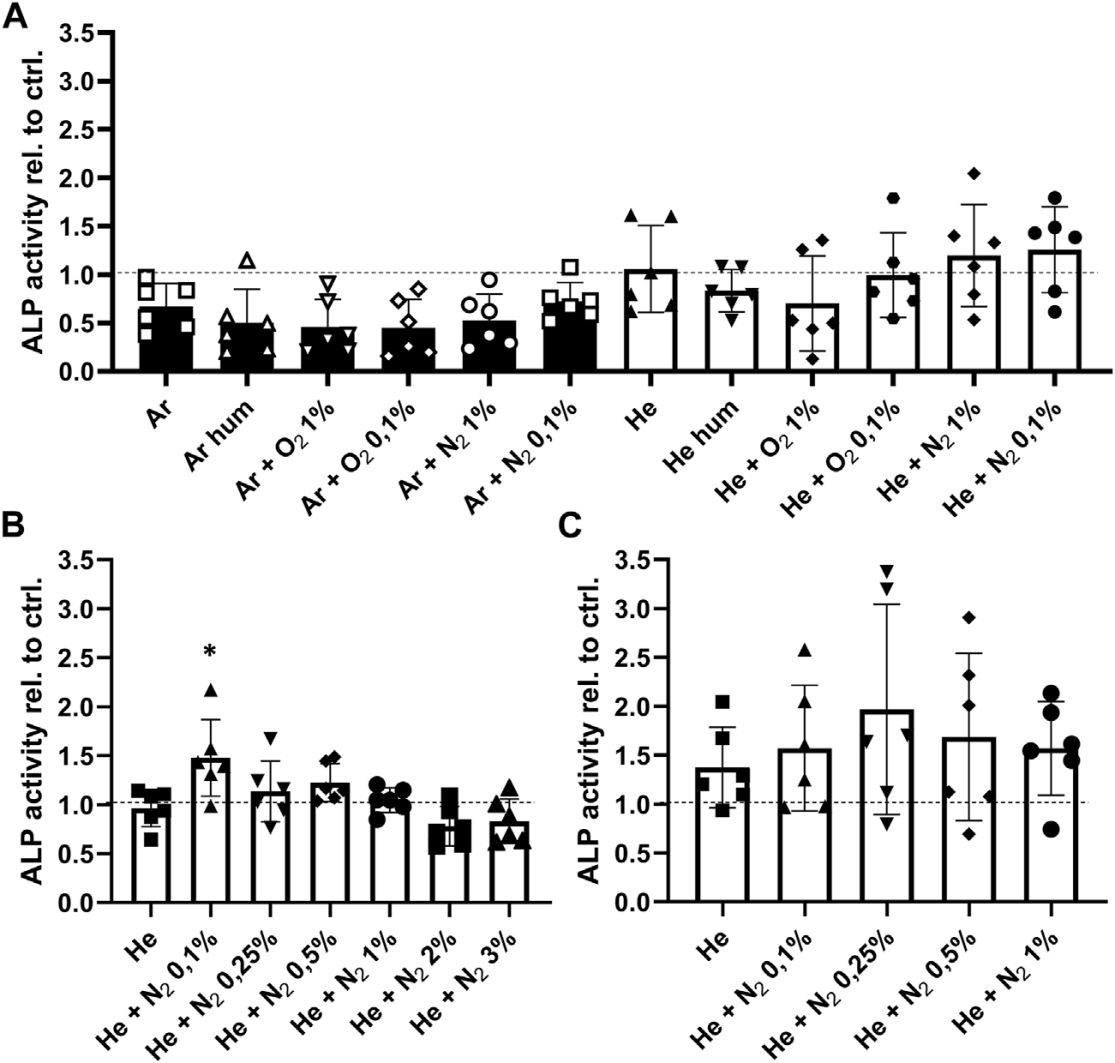
Optimisation of CPP gas source and treatment time to improve osteogenic function of human mesenchymal stromal cells (hMSCs). **(A)** Shown is the ALP activity as a measure of osteogenic function after CPP treatment for 1 min with varying gas sources. **(B)** Since viability was found to be reduced when Ar based gas sources are used, a 3 min treatment time was performed using an He based carrier gas. **(C)** After prolonging the treatment time to 5 min, hMSCs were seeded onto the scaffolds and their ALP activity was determined after 7 days. [**(A–C)** one-way ANOVA, multiple comparisons, *n* = 6, *p < 0.05].

Next, we investigated whether CPP treatment of the bone affected cell ingrowth, survival and function since these parameters influence the overall outcome of allogenic bone transplantation. The effect of CPP treatment on cells and tissues strongly depends on gas composition and treatment time. It was therefore investigated how these parameters affect cell survival and osteogenic function of hMSCs. For this, different gas compositions were used for CPP treatment with a duration of 1, 3 and 5 min of allogenic bone scaffolds prior to cell seeding. Based on a previously established bone model, MSCs were then seeded on the treated scaffolds and cultured for 7 days in total (Schoon et al., 2020). Investigation of metabolic activity after 4 and 7 days revealed that 1 min Ar-based CPP treatment negatively affected cell viability, as indicated by significantly reduced metabolic activity on day 4 and 7 for Ar-based gas mixtures with O_2_. In contrast, He-based CPP treatment of allogenic scaffolds did not significantly alter cell viability, while addition of N_2_ provided the most favourable outcome (Figure 4A). Furthermore, ALP activity was investigated as a marker for matrix mineralization that is vital for bone formation and found to be unchanged for He-based CPP, while using Ar seems to influence this parameter negatively (Figure 5A). Due to its negative influence on cell viability and function, Ar-based CPP was not further investigated in favour of He as a carrier gas. In a next step, a 3-min-long CPP treatment using different He-based gas mixtures with N_2_ were tested. Metabolic and ALP activity were both found to be significantly elevated after 4 and 7 days when scaffolds were treated with He supplemented with 0.1% N_2_ (Figures 4B, 5B). Prolonging the CPP treatment time to 5 min prior to cell seeding did not result in significantly enhanced outcomes in terms of metabolic activity or osteogenic functions (Figures 4C, 5C). In summary, a 3-min CPP treatment with a mixture of He + 0.1% N_2_ had the most favourable outcome in terms of significantly elevated viability and osteogenic function of hMSCs and should thus be further investigated for the processing of allogenic bone grafts rather than utilizing Ar-based CPP.

## 4 Discussion

The aim of this study was to analyze the feasibility of CPP treatment for pre-therapeutic allogeneic bone graft processing by evaluating whether CPP alters morphological bone properties or influences hMSC viability and function. The morphological parameters investigated were Tb.Th, Tb.Sp, TV, BV, BS, and Po. Overall the CPP treatment for 5 min showed no alteration in the analyzed bone parameters by comparison of pre- and post-treatment SRµCT scans. A deviation of morphological parameters between different scaffolds was expected as it is representative of donor and harvest site-dependent differences of bone (Goldman et al., 2003; Donnelly et al., 2012). The treatment with CPP had no measurable influence on the morphological parameters investigated, which is a prerequisite for further implementation of CPP in the processing of allogeneic bone for clinical applications. In addition, we found that optimising the CPP gas source led to significantly better outcomes regarding hMSC viability and osteogenic function. We determined that a 3 min CPP treatment of allogenic bone resulted in elevated metabolic activity of hMSCs, while ALP activity was also enhanced under these conditions. Our data thus emphasized the need for careful optimization of treatment time and carrier gas prior to CPP application in a given context. The current work indicates the positive effects of CPP treatment on allogenic bone and therefore encourages the use of CPP for graft processing. CPP might be employed for sterilization and surface modification to allow for better cell attachment and the addition of biomolecules to enhance the therapeutic value of the allogeneic bone graft (Coelho et al., 2012; Hui et al., 2020a; Tan et al., 2021; Nonnenmacher et al., 2023). It is well described that CPP shows antimicrobial effects against a range of pathogens, including those that are challenging to eradicate due to biofilm formation (Soler-Arango et al., 2019; Benčina et al., 2021; Scholtz et al., 2021). These effects are attributed to the formation of ROS and RNS and a disturbance of cell membrane integrity due to CPP treatment (Pompl et al., 2009). In the context of processing allogeneic bone, CPP might pose a reasonable alternative to established methods, such as irradiation, that are reported to affect the mechanical properties of the graft (Nguyen et al., 2007). Yet, to further prove the suitability of CPP for sterilization, mechanical properties, in addition to morphological ones, need to be investigated for treated allogeneic bone. Surface treatment with CPP has already been utilized on various metallic, synthetic, and natural bone grafts and demonstrated positive effects on the adhesion and proliferation of musculoskeletal cells (Wang et al., 2016; Tominami et al., 2017; Canullo et al., 2018; Wang et al., 2019; Hui et al., 2020b). CPP treatment is reported to temporarily increase the concentration of reactive oxygen species on the surface of tissue culture vessels made of polystyrene (PS), leading to an increased overall wettability. In addition, CPP treatment is also reported to change the overall charge of PS surfaces, which facilitates the adsorption of proteins that increase cell adhesion (Lerman et al., 2018). Both mechanisms might also be beneficial for the surface activation of allogeneic bone grafts, as an increase in wettability was reported for various implant materials (Duske et al., 2012b; Karaman et al., 2018a; Yang et al., 2018). Due to the low temperature in which CPP operates, coating of biomolecules is also possible using this technique and would allow for directed surface modifications (Yoshinari et al., 2011; Tan and Al-Rubeai, 2019; O'Neill et al., 2021). For example, CPP-assisted coating with proteins and peptides such as collagen I or Arg-Gly-Asp (RGD) peptides has been shown to increase the proliferation of musculoskeletal cells on metallic and polymeric surfaces (Mörke et al., 2017; Karaman et al., 2018b). Therefore, CPP treatment of allogenic bone has various potential applications. Yet, the effect of CPP on cellular ingrowth, viability and function would need further research prior its implementation for allogenic bone processing. The optimization of gas source and treatment time would also need careful consideration and should be followed by morphological investigations using the exact conditions that match later applications with the aim of enhanced cellular response. Albeit the present study demonstrates overall feasibility of CPP treatment for allogenic bone scaffolds, it is limited by not including investigations on the topographical and structural level. For investigation of overall topography that also impacts the osseointegration of implants, methods that provide high spatial accuracy such as scanning electron microscopy or confocal laser scanning microscopy should be employed (Hou et al., 2022; Liu et al., 2023). On the structural level, investigations in terms of integrity and organization of collagen fibrils should be performed by using methods such as Raman confocal microscopy or second harmonic imaging (Chen et al., 2012; Schrof et al., 2014; Couture et al., 2015). Furthermore, it would need to be asserted that the immunogenicity profile of the allogenic bone scaffolds is not altered, to avoid rejection of the graft by the host (Huzum et al., 2021; Sharifi et al., 2022; Kasravi et al., 2023). In summary, CPP treatment of allogenic bone has high potential to improve clinical outcomes after grafting but needs further investigations regarding topography, structure and immunogenicity.

## 5 Conclusion

CPP treatment did not alter the morphological properties of allogeneic bone grafts and might therefore be employed for processing prior to their clinical use. In addition, careful optimization of the employed carrier gas can help to enhance the biological response after grafting and thus the clinical performance. Due to the many advantageous characteristics of CPP and the possibility for surface modifications, CPP treatment is a promising tool for the processing of allogenic bone.

## Data Availability

The raw data supporting the conclusion of this article will be made available by the authors, without undue reservation.

## References

[B1] Ahmed N. Eras V. Pruß A. Perka C. Brune J. Vu-Han T. L. (2023). Allografts: expanding the surgeon’s armamentarium. Cell Tissue Bank. 24 (1), 273–283. 10.1007/s10561-022-10015-7 35763162 PMC10006263

[B2] Baldwin P. Li D. J. Auston D. A. Mir H. S. Yoon R. S. Koval K. J. (2019). Autograft, allograft, and bone graft substitutes: clinical evidence and indications for use in the setting of orthopaedic trauma surgery. J. Orthop. Trauma 33 (4), 203–213. 10.1097/bot.0000000000001420 30633080

[B3] Bekeschus S. Schmidt A. Weltmann K. D. von Woedtke T. (2016). The plasma jet kINPen – a powerful tool for wound healing. Clin. Plasma Med. 4 (1), 19–28. 10.1016/j.cpme.2016.01.001

[B4] Benčina M. Resnik M. Starič P. Junkar I. (2021). Use of plasma technologies for antibacterial surface properties of metals. Molecules 26, 1418. 10.3390/molecules26051418 33808010 PMC7961478

[B5] Blokhuis T. Lindner T. (2008). Allograft and bone morphogenetic proteins: an overview. Injury 39, S33–S36. 10.1016/s0020-1383(08)70013-5 18804571

[B6] Boyce T. Edwards J. Scarborough N. (1999). Allograft bone: the influence of processing on safety and performance. Orthop. Clin. 30 (4), 571–581. 10.1016/s0030-5898(05)70110-3 10471762

[B7] Brydone A. Meek D. Maclaine S. (2010). Bone grafting, orthopaedic biomaterials, and the clinical need for bone engineering. Proc. Institution Mech. Eng. Part H J. Eng. Med. 224 (12), 1329–1343. 10.1243/09544119jeim770 21287823

[B8] Campana V. Milano G. Pagano E. Barba M. Cicione C. Salonna G. (2014). Bone substitutes in orthopaedic surgery: from basic science to clinical practice. J. Mater. Sci. Mater. Med. 25 (10), 2445–2461. 10.1007/s10856-014-5240-2 24865980 PMC4169585

[B9] Canal C. Khurana K. Gallinetti S. Bhatt S. Pulpytel J. Arefi-Khonsari F. (2016). Design of calcium phosphate scaffolds with controlled simvastatin release by plasma polymerisation. Polymer 92, 170–178. 10.1016/j.polymer.2016.03.069

[B10] Canullo L. Cassinelli C. Götz W. Tarnow D. (2013). Plasma of argon accelerates murine fibroblast adhesion in early stages of titanium disk colonization. Int. J. Oral Maxillofac. Implants 28 (4), 957–962. 10.11607/jomi.2664 23869352

[B11] Canullo L. Genova T. Naenni N. Nakajima Y. Masuda K. Mussano F. (2018). Plasma of argon enhances the adhesion of murine osteoblasts on different graft materials. Ann. Anatomy-Anatomischer Anzeiger 218, 265–270. 10.1016/j.aanat.2018.03.005 29704635

[B12] Chen X. Nadiarynkh O. Plotnikov S. Campagnola P. J. (2012). Second harmonic generation microscopy for quantitative analysis of collagen fibrillar structure. Nat. Protoc. 7 (4), 654–669. 10.1038/nprot.2012.009 22402635 PMC4337962

[B13] Coelho P. G. Giro G. Teixeira H. S. Marin C. Witek L. Thompson V. P. (2012). Argon-based atmospheric pressure plasma enhances early bone response to rough titanium surfaces. J. Biomed. Mater. Res. Part A 100A (7), 1901–1906. 10.1002/jbm.a.34127 22492543

[B14] Couture C. A. Bancelin S. Van der Kolk J. Popov K. Rivard M. Légaré K. (2015). The impact of collagen fibril polarity on second harmonic generation microscopy. Biophys. J. 109 (12), 2501–2510. 10.1016/j.bpj.2015.10.040 26682809 PMC4699883

[B15] DePaula C. A. Truncale K. Gertzman A. Sunwoo M. Dunn M. (2005). Effects of hydrogen peroxide cleaning procedures on bone graft osteoinductivity and mechanical properties. Cell Tissue Bank. 6 (4), 287–298. 10.1007/s10561-005-3148-2 16308768

[B16] Donnelly E. Meredith D. S. Nguyen J. T. Boskey A. L. (2012). Bone tissue composition varies across anatomic sites in the proximal femur and the iliac crest. J. Orthop. Res. 30 (5), 700–706. 10.1002/jor.21574 22034199 PMC3277807

[B17] Duske K. Koban I. Kindel E. Schröder K. Nebe B. Holtfreter B. (2012a). Atmospheric plasma enhances wettability and cell spreading on dental implant metals. J. Clin. Periodontol. 39 (4), 400–407. 10.1111/j.1600-051x.2012.01853.x 22324415

[B18] Duske K. Koban I. Kindel E. Schröder K. Nebe B. Holtfreter B. (2012b). Atmospheric plasma enhances wettability and cell spreading on dental implant metals. J. Clin. Periodontology 39 (4), 400–407. 10.1111/j.1600-051x.2012.01853.x 22324415

[B19] Fillingham Y. Jacobs J. (2016). Bone grafts and their substitutes. Bone & Jt. J. 98-B (1), 6–9. 10.1302/0301-620x.98b.36350 26733632

[B20] Fischer M. Schoon J. Freund E. Miebach L. Weltmann K. D. Bekeschus S. (2022). Biocompatible gas plasma treatment affects secretion profiles but not osteogenic differentiation in patient-derived mesenchymal stromal cells. Int. J. Mol. Sci. 23 (4), 2038. 10.3390/ijms23042038 35216160 PMC8879607

[B21] Fretwurst T. Spanou A. Nelson K. Wein M. Steinberg T. Stricker A. (2014). Comparison of four different allogeneic bone grafts for alveolar ridge reconstruction: a preliminary histologic and biochemical analysis. Oral Surg. Oral Med. Oral Pathol. Oral Radiol. 118 (4), 424–431. 10.1016/j.oooo.2014.05.020 25183228

[B22] Gillman C. E. Jayasuriya A. C. (2021). FDA-approved bone grafts and bone graft substitute devices in bone regeneration. Mater. Sci. Eng. C 130, 112466. 10.1016/j.msec.2021.112466 PMC8555702 34702541

[B23] Goldman H. M. Bromage T. G. Boyde A. Thomas C. D. L. Clement J. G. (2003). Intrapopulation variability in mineralization density at the human femoral mid-shaft. J. Anat. 203 (2), 243–255. 10.1046/j.1469-7580.2003.00212.x 12924824 PMC1571158

[B24] Hadjizadeh A. Doillon C. J. (2010). Directional migration of endothelial cells towards angiogenesis using polymer fibres in a 3D co-culture system. J. Tissue Eng. Regen. Med. 4 (7), 524–531. 10.1002/term.269 20872739

[B25] Hou C. An J. Zhao D. Ma X. Zhang W. Zhao W. (2022). Surface modification techniques to produce micro/nano-scale topographies on Ti-based implant surfaces for improved osseointegration. Front. Bioeng. Biotechnol. 10, 835008. 10.3389/fbioe.2022.835008 35402405 PMC8990803

[B26] Hui W. L. Perrotti V. Iaculli F. Piattelli A. Quaranta A. (2020a). The emerging role of cold atmospheric plasma in implantology: a review of the literature. Nanomaterials 10, 1505. 10.3390/nano10081505 32751895 PMC7466481

[B27] Hui W. L. Perrotti V. Iaculli F. Piattelli A. Quaranta A. (2020b). The emerging role of cold atmospheric plasma in implantology: a review of the literature. Nanomaterials 10 (8), 1505. 10.3390/nano10081505 32751895 PMC7466481

[B28] Huzum B. Puha B. Necoara R. Gheorghevici S. Puha G. Filip A. (2021). Biocompatibility assessment of biomaterials used in orthopedic devices: an overview (Review). Exp. Ther. Med. 22 (5), 1315. 10.3892/etm.2021.10750 34630669 PMC8461597

[B29] Karaman O. Kelebek S. Demirci E. A. İbiş F. Ulu M. Ercan U. K. (2018a). Synergistic effect of cold plasma treatment and RGD peptide coating on cell proliferation over titanium surfaces. Tissue Eng. Regen. Med. 15 (1), 13–24. 10.1007/s13770-017-0087-5 30603531 PMC6171635

[B30] Karaman O. Kelebek S. Demirci E. A. İbiş F. Ulu M. Ercan U. K. (2018b). Synergistic effect of cold plasma treatment and RGD peptide coating on cell proliferation over titanium surfaces. Tissue Eng. Regen. Med. 15 (1), 13–24. 10.1007/s13770-017-0087-5 30603531 PMC6171635

[B31] Kasravi M. Ahmadi A. Babajani A. Mazloomnejad R. Hatamnejad M. R. Shariatzadeh S. (2023). Immunogenicity of decellularized extracellular matrix scaffolds: a bottleneck in tissue engineering and regenerative medicine. Biomaterials Res. 27 (1), 10. 10.1186/s40824-023-00348-z PMC9912640 36759929

[B32] Killinger A. Gadow R. (2021). “Thermally sprayed materials for biomedical applications,” in Encyclopedia of materials: technical ceramics and glasses. Editor Pomeroy M. (Oxford: Elsevier), 732–749.

[B33] Lei P. Sun R. Wang L. Zhou J. Wan L. Zhou T. (2015). A new method for xenogeneic bone graft deproteinization: comparative study of radius defects in a rabbit model. PLoS One 10 (12), e0146005. 10.1371/journal.pone.0146005 26719896 PMC4699924

[B34] Lerman M. J. Lembong J. Muramoto S. Gillen G. Fisher J. P. (2018). The evolution of polystyrene as a cell culture material. Tissue Eng. Part B Rev. 24 (5), 359–372. 10.1089/ten.teb.2018.0056 29631491 PMC6199621

[B35] Liu J. Yang L. Zhang H. Zhang J. Hu Y. (2023). Effects of allogeneic bone substitute configurations on cell adhesion process *in vitro* . Orthop. Surg. 15 (2), 579–590. 10.1111/os.13395 36453151 PMC9891915

[B36] Mansor A. Ariffin A. F. Yusof N. Mohd S. Ramalingam S. Md Saad A. P. (2023). Effects of processing and gamma radiation on mechanical properties and organic composition of frozen, freeze-dried and demineralised human cortical bone allograft. Cell tissue Bank. 24 (1), 25–35. 10.1007/s10561-022-10013-9 35610332

[B37] Mirone A. Brun E. Gouillart E. Tafforeau P. Kieffer J. (2014). The PyHST2 hybrid distributed code for high speed tomographic reconstruction with iterative reconstruction and *a priori* knowledge capabilities. Nucl. Instrum. Methods Phys. Res. Sect. B-Beam Interact. Mater. Atoms 324, 41–48. 10.1016/j.nimb.2013.09.030

[B38] Mörke C. Rebl H. Finke B. Dubs M. Nestler P. Airoudj A. (2017). Abrogated cell contact guidance on amino-functionalized microgrooves. ACS Appl. Mater Interfaces 9 (12), 10461–10471. 10.1021/acsami.6b16430 28296389

[B39] Nguyen H. Morgan D. A. Forwood M. R. (2007). Sterilization of allograft bone: is 25 kGy the gold standard for gamma irradiation? Cell Tissue Bank. 8 (2), 81–91. 10.1007/s10561-006-9019-7 16821106

[B40] Nonnenmacher L. Fischer M. Haralambiev L. Bekeschus S. Schulze F. Wassilew G. I. (2023). Orthopaedic applications of cold physical plasma. EFORT Open Rev. 8 (6), 409–423. 10.1530/eor-22-0106 37289098 PMC10300842

[B41] O'Brien F. J. (2011). Biomaterials & scaffolds for tissue engineering. Mater. Today 14 (3), 88–95. 10.1016/s1369-7021(11)70058-x

[B42] O'Neill L. Twomey B. O'Donoghue J. Hunt J. A. (2021). Collagen coating of titanium implants using nonthermal plasma. Collagen Coat. Titanium Implants Using Nonthermal Plasma 11 (2), 63–79. 10.1615/plasmamed.2021039685

[B43] Otto R. Sørby K. Hesse B. Gerber J. Bortel E. Kiener C. (2022). Synchrotron µ-CT-based morphological characterization of additively manufactured open porous structures. Addit. Manuf. 55, 102874. 10.1016/j.addma.2022.102874

[B44] Paganin D. Mayo S. C. Gureyev T. E. Miller P. R. Wilkins S. W. (2002). Simultaneous phase and amplitude extraction from a single defocused image of a homogeneous object. J. Microsc. 206, 33–40. 10.1046/j.1365-2818.2002.01010.x 12000561

[B45] Pompl R. Jamitzky F. Shimizu T. Steffes B. Bunk W. Schmidt H. U. (2009). The effect of low-temperature plasma on bacteria as observed by repeated AFM imaging. New J. Phys. 11 (11), 115023. 10.1088/1367-2630/11/11/115023

[B46] Rakow A. Schoon J. Dienelt A. John T. Textor M. Duda G. (2016). Influence of particulate and dissociated metal-on-metal hip endoprosthesis wear on mesenchymal stromal cells *in vivo* and *in vitro* . Biomaterials 98, 31–40. 10.1016/j.biomaterials.2016.04.023 27179133

[B47] Ratha I. Datta P. Balla V. K. Nandi S. K. Kundu B. (2021). Effect of doping in hydroxyapatite as coating material on biomedical implants by plasma spraying method: a review. Ceram. Int. 47 (4), 4426–4445. 10.1016/j.ceramint.2020.10.112

[B48] Reuter S. Von Woedtke T. Weltmann K.-D. (2018). The kINPen—a review on physics and chemistry of the atmospheric pressure plasma jet and its applications. J. Phys. D Appl. Phys. 51 (23), 233001. 10.1088/1361-6463/aab3ad

[B49] Rothweiler R. Gross C. Bortel E. Früh S. Gerber J. Boller E. (2022). Comparison of the 3D-microstructure between alveolar and iliac bone for enhanced bioinspired bone graft substitutes. Front. Bioeng. Biotechnol. 10, 862395. 10.3389/fbioe.2022.862395 35782504 PMC9248932

[B50] Rupp M. Klute L. Baertl S. Walter N. Mannala G. Frank L. (2022). The clinical use of bone graft substitutes in orthopedic surgery in Germany—a 10‐years survey from 2008 to 2018 of 1,090,167 surgical interventions. J. Biomed. Mater. Res. Part B Appl. Biomaterials 110 (2), 350–357. 10.1002/jbm.b.34911 34291874

[B51] Sardella E. Salama R. A. Waly G. H. Habib A. N. Favia P. Gristina R. (2017). Improving internal cell colonization of porous scaffolds with chemical gradients produced by plasma assisted approaches. ACS Appl. Mater Interfaces 9 (5), 4966–4975. 10.1021/acsami.6b14170 28094986

[B52] Scheinpflug J. Pfeiffenberger M. Damerau A. Schwarz F. Textor M. Lang A. (2018). Journey into bone models: a review. Genes (Basel) 9 (5), 247. 10.3390/genes9050247 29748516 PMC5977187

[B53] Scholtz V. Vaňková E. Kašparová P. Premanath R. Karunasagar I. Julák J. (2021). Non-thermal plasma treatment of eskape pathogens: a review. Front. Microbiol. 12, 737635. 10.3389/fmicb.2021.737635 34712211 PMC8546340

[B54] Schoon J. Hesse B. Rakow A. Ort M. J. Lagrange A. Jacobi D. (2020). Metal-specific biomaterial accumulation in human peri-implant bone and bone marrow. Adv. Sci. (Weinh) 7 (20), 2000412. 10.1002/advs.202000412 33101844 PMC7578891

[B55] Schrof S. Varga P. Galvis L. Raum K. Masic A. (2014). 3D Raman mapping of the collagen fibril orientation in human osteonal lamellae. J. Struct. Biol. 187 (3), 266–275. 10.1016/j.jsb.2014.07.001 25025981

[B56] Schulze F. Lang A. Schoon J. Wassilew G. I. Reichert J. (2023). Scaffold guided bone regeneration for the treatment of large segmental defects in long bones. Biomedicines 11 (2), 325. 10.3390/biomedicines11020325 36830862 PMC9953456

[B57] Sharifi M. Kheradmandi R. Salehi M. Alizadeh M. ten Hagen T. L. M. Falahati M. (2022). Criteria, challenges, and opportunities for acellularized allogeneic/xenogeneic bone grafts in bone repairing. ACS Biomaterials Sci. Eng. 8 (8), 3199–3219. 10.1021/acsbiomaterials.2c00194 35816626

[B58] Sohn H. S. Oh J. K. (2019). Review of bone graft and bone substitutes with an emphasis on fracture surgeries. Biomater. Res. 23, 9. 10.1186/s40824-019-0157-y 30915231 PMC6417250

[B59] Soler-Arango J. Figoli C. Muraca G. Bosch A. Brelles-Mariño G. (2019). The *Pseudomonas aeruginosa* biofilm matrix and cells are drastically impacted by gas discharge plasma treatment: a comprehensive model explaining plasma-mediated biofilm eradication. PLoS One 14 (6), e0216817. 10.1371/journal.pone.0216817 31233528 PMC6590783

[B60] Tan F. Al-Rubeai M. (2019). Customizable implant-specific and tissue-specific extracellular matrix protein coatings fabricated using atmospheric plasma. Front. Bioeng. Biotechnol. 7, 247. 10.3389/fbioe.2019.00247 31637236 PMC6787931

[B61] Tan F. Fang Y. Zhu L. Al-Rubeai M. (2021). Cold atmospheric plasma as an interface biotechnology for enhancing surgical implants. Crit. Rev. Biotechnol. 41 (3), 425–440. 10.1080/07388551.2020.1853671 33622112

[B62] Tominami K. Kanetaka H. Sasaki S. Mokudai T. Kaneko T. Niwano Y. (2017). Cold atmospheric plasma enhances osteoblast differentiation. PLoS One 12 (7), e0180507. 10.1371/journal.pone.0180507 28683076 PMC5500351

[B63] von Woedtke T. Emmert S. Metelmann H. R. Rupf S. Weltmann K. D. (2020). Perspectives on cold atmospheric plasma (CAP) applications in medicine. Phys. Plasmas 27 (7), 070601. 10.1063/5.0008093

[B64] Wang M. Favi P. Cheng X. Golshan N. H. Ziemer K. S. Keidar M. (2016). Cold atmospheric plasma (CAP) surface nanomodified 3D printed polylactic acid (PLA) scaffolds for bone regeneration. Acta Biomater. 46, 256–265. 10.1016/j.actbio.2016.09.030 27667017

[B65] Wang M. Zhou Y. Shi D. Chang R. Zhang J. Keidar M. (2019). Cold atmospheric plasma (CAP)-modified and bioactive protein-loaded core-shell nanofibers for bone tissue engineering applications. Biomater. Sci. 7 (6), 2430–2439. 10.1039/c8bm01284a 30933194

[B66] Weitkamp T. Scheel M. Giorgetta J. Joyet V. Le Roux V. Cauchon G. (2017). The tomography beamline ANATOMIX at Synchrotron SOLEIL. J. Phys. Conf. Ser. 849 (1), 012037. 10.1088/1742-6596/849/1/012037

[B67] Weitkamp T. Scheel M. Perrin J. Daniel G. King A. Le Roux V. (2022). Microtomography on the ANATOMIX beamline at synchrotron SOLEIL. J. Phys. Conf. Ser. 2380 (1), 012122. 10.1088/1742-6596/2380/1/012122

[B68] Weltmann K. D. Kindel E. von Woedtke T. Hähnel M. Stieber M. Brandenburg R. (2010). Atmospheric-pressure plasma sources: prospective tools for plasma medicine. Atmospheric-pressure plasma sources Prospect. tools plasma Med. 82 (6), 1223–1237. 10.1351/pac-con-09-10-35

[B69] Yang Y. Guo J. Zhou X. Liu Z. Wang C. Wang K. (2018). A novel cold atmospheric pressure air plasma jet for peri-implantitis treatment: an <i&gt;*in vitro*&lt;/i&gt; study. Dent. Mater. J. 37 (1), 157–166. 10.4012/dmj.2017-030 29176301

[B70] Yoshinari M. Matsuzaka K. Inoue T. (2011). Surface modification by cold-plasma technique for dental implants—bio-functionalization with binding pharmaceuticals. Jpn. Dent. Sci. Rev. 47 (2), 89–101. 10.1016/j.jdsr.2011.03.001

